# EEG-Based ADHD Classification Using Autoencoder Feature Extraction and ResNet with Double Augmented Attention Mechanism

**DOI:** 10.3390/brainsci15010095

**Published:** 2025-01-20

**Authors:** Jayoti Bansal, Gaurav Gangwar, Mohammad Aljaidi, Ali Alkoradees, Gagandeep Singh

**Affiliations:** 1 Department of Computer Science Engineering, Baba Farid College of Engineering & Technology, Bathinda 151001, Punjab, India; gauravwar04@gmail.com; 2 Department of Computer Science, Zarqa University, Zarqa 13110, Jordan; mjaidi@zu.edu.jo; 3 Unit of Scientific Research, Applied College, Qassim University, Buraydah 52571, Saudi Arabia; alifk@qu.edu.sa; 4 Department of Mechanical Engineering, Baba Farid College of Engineering & Technology, Bathinda 151001, Punjab, India; gagan.1804@gmail.com

**Keywords:** ADHD, ResNet, double augmented attention mechanism, EEG, auto encoder, reptile search algorithm

## Abstract

Background: Attention-Deficit/Hyperactivity Disorder (ADHD) represents a widely prevalent and heterogeneous neurodevelopmental condition in pediatric populations, often exhibiting a substantial propensity to persist into adulthood. ADHD is a multifaceted disorder that resists straightforward diagnostic tests. Clinicians must invest substantial time and effort to secure an accurate diagnosis and implement effective treatment. ADHD diagnosis is primarily based on psychiatric tests, as there is currently no clinically utilized objective diagnostic tool. Nonetheless, several studies in have documented endeavors to create objective instruments designed to assist in the diagnostic process of ADHD, aiming to enhance diagnostic accuracy and reduce subjectivity. Method: This research endeavor sought to establish an objective diagnostic modality for ADHD through the utilization of electroencephalography (EEG) signal analysis. With the use of innovative deep learning techniques, this research seeks to improve the diagnosis of ADHD using EEG data. To capture complex patterns in EEG data, this study proposes a double-augmented attention mechanism ResNet-based model. Using an autoencoder for feature extraction, the Reptile Search Algorithm for feature selection, and a modified ResNet architecture for model training comprise the technique. Results: AUC, F1-score, accuracy, precision, recall, and other standard classifiers like Random Forest and AdaBoost were utilized to compare the model’s performance. By a wide margin, the proposed ResNet model outperforms the traditional models with a 99.42% accuracy, 99.03% precision, 99.82% recall, and 99.42% F1-score. Conclusions: ROC AUC score of 0.99 for the model underscores its remarkable capability to differentiate between children with and without ADHD, thereby minimizing misclassification errors and improving diagnostic precision.

## 1. Introduction

Attention deficit hyperactivity disorder (ADHD) is a common disorder in children, marked by ongoing issues with focus, easy distraction, hyperactivity, and impulsive behavior [1]. ADHD affects individuals from childhood through adulthood, with children and adolescents making up over 80% of the total cases. Moreover, the number of children and teenagers diagnosed with ADHD rises progressively each year [2]. If ADHD is not treated, its symptoms can cause ongoing challenges for children as they grow up [3]. Children with ADHD have a 70% chance of still having it in their teen years, and if it is not treated, it can continue into adulthood in more than half of the cases. The prompt ascertainment and initiation of therapeutic measures for ADHD are crucial, given their profound impact on alleviating the disorder’s pervasive developmental, cognitive, and social ramifications [4,5]. The wide range of symptoms and the common presence of other conditions with ADHD make it hard for doctors to diagnose it accurately, which can lead to missed cases or mistaken diagnoses [6,7]. Missed diagnoses leave patients dealing with the harmful effects of the disorder, while erroneous diagnoses result in unsuitable therapeutic interventions, potentially accompanied by adverse side effects. Delays in accurate diagnosis and treatment can lead to higher healthcare costs utilization, often driven by the worsening of other mental and physical health conditions that occur together [8,9].

While conventional statistical techniques can assess predictive accuracy, they often fall short in capturing intricate, non-linear relationships, especially when numerous predictive variables interact to shape outcomes. Conversely, machine learning can adeptly navigate these complexities, assuming that an adequately large dataset is available [10]. Although machine learning has been employed on objective datasets, its application is restricted by the substantial expense associated with data acquisition, thereby limiting the breadth of accessible sample sizes. Furthermore, small sample sizes can result in inflated accuracy estimates if machine learning techniques are incorrectly applied [11,12,13].

Since 2010, there has been active research on diagnosing ADHD through artificial intelligence, capitalizing on the advancements in machine learning and deep learning technologies. The research is increasingly centered on creating faster and more efficient methods for diagnosing ADHD. This entails leveraging a diverse array of objective data sources, including accelerometer readings, simulation results, and interactive gaming outputs, in conjunction with biometric metrics such as MRI, EEG, and ECG, to augment diagnostic accuracy and efficacy. These findings are expected to augment the precision of ADHD diagnoses and streamline the temporal demands associated with this diagnostic process. Specifically, the dissemination of the Neuro Bureau ADHD-200 dataset has facilitated the advancement of research by providing a comprehensive and standardized repository of data for the investigation of ADHD, which has prompted various research institutes to actively investigate methods for improving ADHD diagnosis accuracy. Their efforts are concentrated on formulating advanced machine learning and deep learning algorithms utilizing this dataset [14].

In assessment, electroencephalogram (EEG) tracking of brain activity is quicker, less expensive, more transportable, and more descriptive. Therefore, electroencephalogram (EEG) may be a beneficial tool for studying and assessing the peculiar functioning of children with attention deficit hyperactivity disease (ADHD). Further studies on using EEG testing to diagnose ADHD are important to enhance the modern-day State of the Art and provide more accurate results. Lots of records are included within the indicators of an electroencephalogram (EEG). Manual identification of outliers is not much better either. This is the optimal point for machine learning (ML). The basic idea behind machine learning is to train computers to improve a performance metric using previous work or example data; this technique might be applied to the current task. In order to improve the accuracy of ADHD (Attention-Deficit/Hyperactivity Disorder) classifications using EEG data, the research introduces a novel technique. By combining advanced feature extraction methods and a complex classification system, the study aims to address the challenges of accurately diagnosing ADHD.

The main contributions of the study are as follows:

- This study introduces a novel method for extracting meaningful features from EEG data using auto-encoders, effectively reducing noise and improving data entry quality for ADHD designation.
- A deep residual network (ResNet) is used for classification, which offers the advantage of being able to control fluid loss and improve model performance on complex EEG datasets.
- The study integrates an advanced dual attention mechanism to selectively prioritize and emphasize the most pertinent features, thereby enhancing the model’s ability to capture critical patterns, dramatically enhancing the model’s sensitivity and accuracy in distinguishing between non-ADHD and ADHD.

## 2. Literature Review

Electroencephalography (EEG) is a non-invasive method for monitoring and recording electrical signals and voltage fluctuations generated by neuronal activity in the brain, using electrodes attached to the scalp. EEG can record brain activity over extended periods and measure neural signals, providing valuable information for analyzing and treating health issues related to brain function, which serves as a crucial data source for various fields, including neuroscience, biomedical engineering, and brain–computer interface studies. Its accessibility makes it valuable for numerous neuroscience research applications [15,16]. Chen M. (2019) conducted a retrospective case–control study that leveraged pre-existing data from the Neuro Bureau ADHD-200 dataset, encompassing 973 participants. Multiscale functional brain connectomes were constructed, integrating both anatomical and functional criteria to capture complex brain connectivity patterns. During cross-validation, the mcDNN model—utilizing an integrated feature set combining multiscale brain connectome data and Principal Component Decomposition (PCD)—demonstrated superior accuracy in ADHD detection, achieving an AUC of 0.82 (95% CI: 0.80, 0.83), thereby surpassing scDNN models, which analyzed features from each brain connectome scale and PCD independently. In hold-out validation, the mcDNN model maintained robust performance, yielding an AUC of 0.74 (95% CI: 0.73, 0.76) [17]. In 2020, Dubreuil-Vall presented a four-layer convolutional neural network (CNN) architecture, integrating filtering and pooling operations. The model was trained on stacked multi-channel EEG time-frequency decompositions (spectrograms) derived from event-related potentials (ERPs) in ADHD patients (n = 20) and healthy controls (n = 20), recorded during the Flanker Task, with 2800 samples per group. This approach mirrors techniques used in audio and image classification, employing deep neural networks to autonomously identify invariant and compositional features within the data. Achieving a classification accuracy of 88% ± 1.12%, the model outperformed both Recurrent Neural Networks and Shallow Neural Networks, obviating the need for manual feature selection based on EEG spectral or channel-specific characteristics [18]. Tosun (2021) analyzed the effects of photic stimuli at various frequencies and across different channels on ADHD diagnosis, aiming to identify the most effective channel and recording state for accurate ADHD detection. The dataset, obtained using power spectral densities and spectral entropy values from individuals with and without ADHD, was used in experiments with long short-term memory (LSTM), support vector machine (SVM), and artificial neural network classifiers. Among these, LSTM achieved the highest accuracy, with 88.88% accuracy on the “Fp1, F7” channel and 92.15% in the eyes-closed resting state. Spectral entropy was found to enhance classification accuracy positively [19]. Zhou D (2021) summarized the brain electrical activity and clinical characteristics of children aged between 6 and 16 with ADHD in a children’s hospital, using long-range EEG video data to assess their clinical diagnostic significance. Deep learning models, including fully connected neural networks, 2D convolutional neural networks, and LSTM networks, were employed, achieving an average accuracy of 97.7% and a false negative rate of 2.2%. The results highlight the strong generalization capability of the convolutional neural network in accurately identifying ADHD in each participant [20]. Donglin Wang (2022) describes a method that combines independent component analysis (ICA) with a convolutional neural network (CNN), where ICA extracts independent components from each subject, which are then fed into a CNN to classify ADHD patients from typical controls. The second method, the correlation autoencoder, uses brain region correlations as input to an autoencoder, learning latent features for classification through a new neural network. Although each method captures inter-voxel information from fMRI differently, both employ CNNs to extract predictive features. These methods achieved an accuracy of around 69% [21]. Ning Qiang (2022) proposes a spatiotemporal attention auto-encoder (STAAE) to identify global features that address learning difficulties in volumetric resting-state fMRI (rfMRI). The unsupervised STAAE framework spatiotemporally models rfMRI sequences, decomposing them into spatial and temporal patterns. While spatial patterns, known as resting-state networks (RSNs), have been extensively studied, temporal patterns have been largely overlooked in recent decades. For ADHD classification, the proposed RSTT-based classification framework achieved a high accuracy of 72.5%, outperforming methods from recent studies [22]. Jungpil (2021) applied a deep learning (DL)-based algorithm that was implemented to distinguish children with ADHD co-occurring with ASD, utilizing functional near-infrared spectroscopy (fNIRS) signals. Data acquisition involved thirteen children diagnosed with ADHD and coexisting ASD, along with fifteen typically developing (TD) children. They performed periodic line (PL) and zigzag line (ZL) drawing tasks under both prediction and tracing conditions, which each task repeated three times. A sophisticated hybrid model, integrating convolutional neural networks (CNN) with bidirectional long short-term memory (Bi-LSTM), was engineered to effectively classify ADHD-ASD comorbidity. This model achieved a classification accuracy of 94.0%, sensitivity of 89.7%, specificity of 97.8%, an F1-score of 93.3%, and an AUC of 0.938, demonstrating robust performance in accurately identifying ADHD with coexisting ASD [23]. Mustafa Yasin Esas et al. explored the efficacy of EEG in detecting ADHD within a clinical cohort, where EEG data were acquired from 121 children aged 7–12, comprising 61 from the ADHD group and 60 from the healthy control group. The study sought to establish an objective diagnostic instrument for ADHD by leveraging EEG signals. These signals were subjected to decomposition into sub-bands utilizing advanced techniques, such as robust local mode decomposition and variational mode decomposition. These sub-bands, along with the original EEG signals, were then input into a custom-designed deep learning algorithm. The resulting algorithm achieved over 95% accuracy in distinguishing ADHD from healthy individuals using 19-channel EEG signals, with a classification accuracy of over 87% through the proposed EEG signal decomposition and processing approach [24]. Miguel Garcia-Argibay et al. (2023) collected data from 238,696 individuals born and residing in Sweden between 1995 and 1999 to assess the effectiveness of various machine learning techniques in aiding ADHD diagnosis in children and adolescents. The methodologies employed encompassed logistic regression, random forest, gradient boosting, XGBoost, penalized logistic regression, deep neural networks (DNN), and ensemble techniques. Of these, the DNN demonstrated superior performance, attaining an area under the receiver operating characteristic curve (AUC) of 0.75 (95% CI: 0.74–0.76) and a balanced accuracy of 0.69. With a probability threshold set at 0.45, the model’s sensitivity was 71.66%, and specificity reached 65.0%, demonstrating its potential in accurately differentiating ADHD cases [25]. Wonjun (2023) developed a game-based system for ADHD screening and diagnosis in children, utilizing five Azure Kinect units with depth sensors to capture skeleton data during gameplay. The skeleton data were divided into standby data, collected while the child observed the robot demonstrating the path, and game data, collected while the child followed the path. Classification was conducted utilizing RNN-based architectures (GRU, RNN, and LSTM), incorporating a bidirectional layer and a weighted cross-entropy loss function. The LSTM model, featuring the bidirectional layer and weighted cross-entropy, attained a classification accuracy of 97.82%, whereas both the GRU and RNN models achieved accuracies of 96.81% [26].

Despite the advancements in ADHD classification using EEG data, several research gaps remain unaddressed. These include the limited exploration of advanced deep learning models, underutilization of feature extraction techniques, and a lack of emphasis on attention mechanisms. Traditional machine learning classifiers and neural networks are the primary methods used, while advanced deep learning architectures like ResNet and attention mechanisms could enhance classification accuracy. Additionally, attention mechanisms are not prioritized in prioritizing the most relevant features in EEG data, which could further refine ADHD classification models’ accuracy and sensitivity. Table 1 shows the datasets used by various researchers for their research on ADHD.

## 3. Methodology

**Figure 1:**
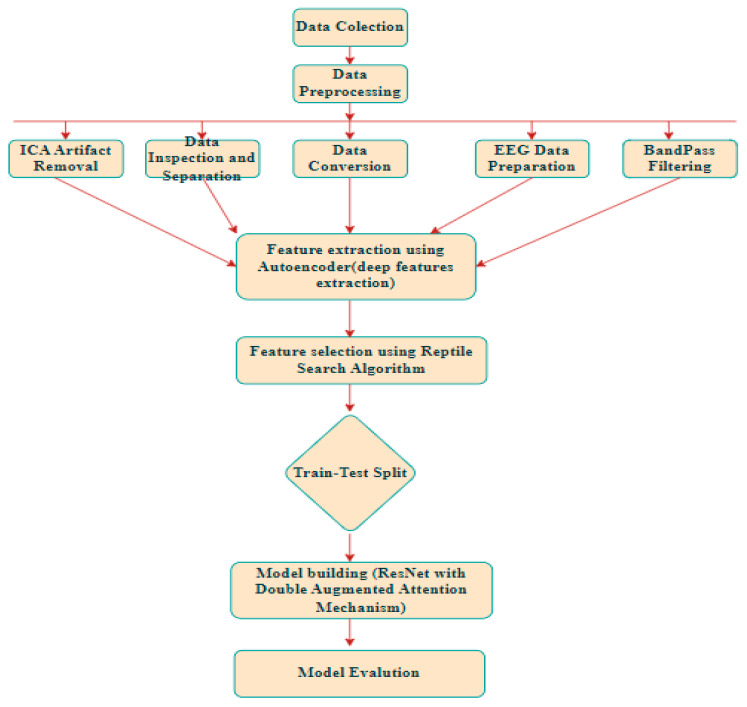
Methodology Flowchart.

In this work, a cutting-edge deep learning algorithm is used to create a unique method for classifying ADHD using EEG data. The methodology is as follows. First, the EEG data are gathered and preprocessed; then, an autoencoder is used to extract deep features; next, the Reptile Search Algorithm is applied to select features; and finally, a ResNet model enhanced with a double-augmented attention mechanism is used for classification. This segment outlines each degree of the method, emphasizing the strategies used to achieve dependable function illustration and high category accuracy. Figure 1 shows the flowchart of methodology.

### 3.1. Data Collection

The EEG dataset for this study was obtained from the IEEE-DataPort database and comprised 121 participants, including 61 individuals diagnosed with ADHD and 60 healthy controls (boys and girls, ages 7–12). The ADHD children were diagnosed by an experienced psychiatrist according to DSM-IV criteria and took Ritalin for up to 6 months. None of the children in the control group had a history of psychiatric disorders, epilepsy, or any report of high-risk behaviors. EEG recording was performed based on 10-20 standard by 19 channels (Fz, Cz, Pz, C3, T3, C4, T4, Fp1, Fp2, F3, F4, F7, F8, P3, P4, T5, T6, O1, O2) at 128 Hz sampling frequency. The A1 and A2 electrodes [27] were the references located on earlobes.

Since one of the deficits in ADHD children is visual attention, the EEG recording protocol was based on visual attention tasks. In the task, a set of pictures of cartoon characters was shown to the children, and they were asked to count the characters. The number of characters in each image was randomly selected between 5 and 16, and the size of the pictures was large enough to be easily visible and countable by children. To have a continuous stimulus during the signal recording, each image was displayed immediately and uninterrupted after the child’s response. Thus, the duration of EEG recording throughout this cognitive visual task was dependent on the child’s performance (i.e., response speed). The recorded dataset consists of 50,000 rows and 20 columns, containing EEG signals recorded through 19 electrodes that depict the 10–20 international system. In this way, each row represents one sample while each column represents one electrode channel where the values consist of the voltage at any given time point from the brain area connected to that electrode. To optimize the data for feature extraction and classification, pre-processing techniques, including noise reduction and artifact removal, were applied.

### 3.2. Data Pre-Processing

In this step, the data were pre-processed in order to prepare the EEG signals ready for analysis by filtering and artifact removal. The following steps ensured the pre-processing followed in this study.

#### 3.2.1. Data Inspection and Separation

The dataset was examined to validate class distribution, differentiating between the ‘normal’ and ‘ADHD’ categories. The data from each class were isolated and reviewed, with class labels saved separately.

#### 3.2.2. EEG Data Preparation

The channel names were retrieved, and the MNE library was used to generate an info object, including EEG channel data and sample frequency. This information item was presented using the conventional 10–20 system montage.

#### 3.2.3. Data Conversion

The EEG data from the normal and ADHD groups were extracted and transformed from DataFrame to NumPy arrays. These arrays were transposed so that channels became rows and samples became columns, and were then converted into MNE RawArray objects.

#### 3.2.4. BandPass Filtering

A bandpass filter was used to keep the frequencies between 4 and 40 Hz. Power Spectral Density (PSD) plots were made both before and after filtering to assess the filter’s effectiveness.

#### 3.2.5. Independent Component Analysis (ICA)

Artifacts in EEG facts were diagnosed and removed from the use of ICA. The EEG data were cleaned by identifying and removing ICA components associated with artifacts. Plotting the cleaned records allowed us to affirm that the artifacts have been successfully removed. Multiple procedures were conducted to validate Independent Component Analysis (ICA) artifact reduction in EEG data. Visual inspection of the original EEG signals and the cleaned signals confirmed that artifacts such eye blinks and muscle movements were removed. SNR and power spectrum analysis were also employed to evaluate signal quality improvement. The SNR increased after artifact removal, indicating higher signal clarity. Additionally, subjective judgment by a trained expert maintained key brain activity and deleted unnecessary artifacts. ICA preprocessing enhanced event-related potential (ERP) and frequency band analysis accuracy and clarity, proving artifact removal’s efficacy.

### 3.3. Train-Test Split

The train-test split is a machine learning technique that splits the dataset into two subsets: the training set and the test set. This is used to analyze the performance of a given model. The model performs well on the training data, and the generalizability of the model toward new data is tested, which is kept separate. Overfitting is when a model does well on the training data but not on fresh, unseen data, and this division avoids it. The most commonly used split ratio is 80/20, where training uses 80% of the data and testing uses the remaining 20%. The proposed method in this study may provide an approximate estimate of the model’s performance by splitting the test set from the training set. This is usually performed using the train test split method in sklearn model selection. The random state parameter within this method ensures that the split is repeatable when running the code, whereas the test size parameter provides the percentage of data to be used for testing. The dataset for this study comprised 50,000 samples. From this dataset, 80% was used for training the model, and the remaining 20% was used for model testing.

### 3.4. Feature Extraction Using Autoencoder

After preprocessing the EEG information, an autoencoder become used to extract deep functions from the alerts, reducing dimensionality while taking pictures complex patterns. This method generated latent capabilities that represented the facts underlying structure, encapsulating excessive-level abstractions and complicated interactions inside EEG signals. Concurrently, time-area parameters inclusive of imply, variance, and skewness had been retrieved, as well as frequency-domain functions like strength spectral density (PSD) and spectral entropy. The autoencoder’s deep functions were then concatenated with the time-area and frequency-domain capabilities, yielding a unified characteristic set that captures each an appropriate shape of the EEG signals and their temporal and spectral houses [28].

### 3.5. Autoencoder Structure

**Figure 2:**
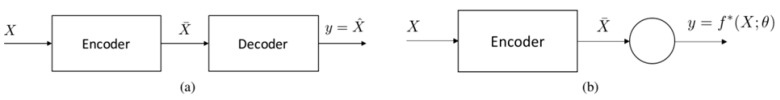
(**a**) Structure of an autoencoder (**b**) Encoder employed in the final estimation.

The architectural decisions of the autoencoder and the role of its latent space in classification can be elaborated for clarity. The autoencoder consists of an encoder and decoder, with the encoder featuring three fully connected layers containing 128, 64, and 32 neurons, respectively, and using the ReLU activation function to capture non-linearity and dynamic EEG patterns effectively. These layer sizes progressively reduce the dimensionality of the input, focusing on extracting meaningful features while discarding noise. The latent space, represented by the bottleneck layer with 32 neurons, encapsulates the most significant aspects of the EEG signals, serving as a compact and highly informative feature representation. This latent space plays a critical role in classification, as it captures high-level abstractions that are fed into the Reptile Search Algorithm for feature selection and subsequently into the ResNet classifier. Further details on how the latent features contribute to specific aspects of classification performance, such as sensitivity to temporal or spatial variations in the EEG data, would enhance understanding. The decoder replicated this structure, recreating the input from the latent space [29]. An autoencoder is symmetrical in design and has two main parts (as shown in Figure 2a,b).

Encoder: An autoencoder for EEG feature extraction generally comprises an encoder that diminishes the dimensionality of the input data. The encoder may comprise multiple fully linked layers or convolutional layers when dealing with spatio-temporal data. The quantity of layers is contingent upon the intricacy of the data and the magnitude of the input features.

Bottleneck Layer: The bottleneck layer, including 32 neurons, served as the latent representation, the latent space extracts the most significant features by eliminating noise and superfluous variations in the data. The latent space improves computational efficiency by ensuring that only the most important features are sent to the classifier by lowering the input dimensions. The network was trained utilizing the Mean Squared Error (MSE) loss function to reduce the reconstruction error between the input and output signals. Mean Squared Error (MSE) was selected for its straightforwardness and efficacy in assessing reconstruction correctness by penalizing significant discrepancies. Alternative loss functions, including Mean Absolute Error (MAE) and Huber loss, were evaluated but found to be inadequate due to their diminished sensitivity to significant mistakes, which are crucial in EEG signal reconstruction [30].

Decoder: The decoder reconstructs the initial input from the latent representation. This component replicates the encoder in reverse, restoring the dimensionality to its original form.

### 3.6. Reptile Search Algorithm

The Reptile search algorithm is a metaheuristic method that is based on how crocodiles naturally hunt [31]. Two phases are necessary for the RSA to function: the hunting phase and the encircling phase. Such an algorithm ensures optimal balances between exploration and exploitation stages: it breaks up into four discernible phases when drawing, making it both suitable and efficient for facing all possible optimization challenges, using similar tactics to those employed by strategic crocodiles hunting. The complete stepwise structure of the Reptile Search Algorithm is given below as Algorithm 1.
**Algorithm 1** Reptile Search Algorithm (RSA)1: **Input:**Population size *N*, maximum iterations *T*, search spacebounds *LB*, *UB* 2: **Initialize** population *P* = {*X*_1_, *X*_2_, …, *X_N_*} randomly within [*LB*,*UB*] 3: **Initialize** bestsolution *X_best_* and its fitness *f_best_* 4: **for** each individual *X_i_* inpopulation *P*
**do** 5: Calculate fitness *f_i_* = *f*(*X_i_*) 6: **if**
*f_i_* < *f_best_*
**then** 7: Update *X_best_* = *X_i_* 8: Update *f_best_* = *f_i_* 9: **end if** 10: **end for** 11: **for** iteration *t* = 1 to *T*
**do** 12: **for** each individual *X_i_* in population *P*
**do** 13: Generate a movement vector *V_i_* based on reptile-inspired strategies 14: Update position *X^new^* = *X_i_* + *V_i_* 15: Apply boundary constraints on *X^new^* to keep with in [*LB*,*UB*] 16: Calculate fitness *f^new^* = *f_i_*(*X_i_^new^*) 17: **if**
*f^new^* < *f_i_*
**then** 18: Accept The New Position *X_i_ = X_i_^new^* 19: Update fitness *f_i_* = *f_i_^new^* 20: **end if** 21: **if**
*f_i_* < *f_best_*
**then** 22: Update *X_best_* = *X_i_* 23: Update *f_best_* = *f_i_* 24: **end if** 25: **end for** 26: Optionally: Apply reptile-specific strategies like warm-up or local search 27: **end for** 28: **Output:** Best solution *X_best_* and its fitness *f_best_*

#### 3.6.1. Initialization

In the first step of the reptile search algorithm, a collection of potential beginning solutions is generated stochastically utilizing the following equation:

(1) $$x_{jk}=rand\times \left(U_{b}-L_{b}\right)+L_{b}\;\;k\;=\;1, 2,\;\ldots ,\;n,$$

where x_jk_ = initialization matrix, j = 1, 2, …, P. P represents population size (rows of the initialization matrix), and n represents the dimensions (columns of the initialization matrix) of the given optimization problem. L_b_, U_b_, and rand represent the lower bound limit, upper bound limit, and randomly generated values [32].

#### 3.6.2. Encircling Phase (Exploration)

During the surrounding phase, densely populated areas are primarily explored. High walking along with belly walking, particularly those derived from crocodile motions, are crucial during the encircling period. These motions aid in finding a large search area but do not contribute to capturing prey.

(2) $$x_{jk}\left(\tau +1\right)=Best_{k}\left(\tau \right)\times \left(-\mu_{\left(jk\right)}\left(\tau \right)\right)\times \beta -\left(R_{\left(jk\right)}\left(\tau \right)\times rand\right),\;T\leq \frac{T}{4},$$

(3) $$x_{jk}\left(\tau +1\right)=Best_{k}\left(\tau \right)\times x_{\left(r_{1},k\right)}\times ES\left(\tau \right)\times rand,\;\tau \leq 2\frac{T}{4}\;and\;\tau >\frac{T}{4},$$

where $Best_{k}\left(\tau \right)$ denotes the optimal solution achieved at the kth position within the current iteration. Here, rand signifies a stochastic variable representing a randomly generated number, while τ represents the current iteration count, with T defining the upper limit for total iterations. The parameter µ(j,k) encapsulates the value of the hunting operator for the jth solution, specifically at the kth position. The calculation of the value for (j,k) is governed by the following formula:

(4) $$\mu_{\left(j,k\right)}=Best_{k}\left(\tau \right)\times P_{\left(j,k\right)},$$

(5) $$R_{\left(j,k\right)}=\frac{Best\left(\tau \right)-P_{\left(r_{2},k\right)}}{Best_{k}\left(\tau \right)+\in },$$

where r_1_ represents a random number selected between 1 and N. Here, N denotes the total number of candidate solutions. The pair (r_1_, l) indicates a random position for the kth solution. Similarly, r_2_ is another randomly chosen number within the range from 1 to N, and ϵ represents a small magnitude. ES(τ), or Evolutionary Sense, is defined as a probability-based ratio that quantifies the evolutionary dynamics within the system. The mathematical formulation of the Evolutionary Sense is given as follows:

(6) $$ES\left(\tau \right)=2\times r_{3}\times \left(1-\frac{1}{T}\right).$$

### 3.7. Feature Selection Using Reptile Search Algorithm

The Reptile seek technique became used to extract the maximum important traits from a huge characteristic collection. Reptile, a meta-mastering method, improves function selection via schooling the version on several subsets of capabilities and improving its overall performance. This iterative process successfully discovers and maintains the maximum critical tendencies, growing version performance and accuracy. The chosen traits were then utilized to educate the final model type.

### 3.8. Model Building

After feature extraction and choice, the next level was to construct a sophisticated classification model making use of ResNet and a double-augmented interest mechanism. The ResNet design, cited for its residual mastering skills, serves to alleviate the vanishing gradient problem and allows for successful deep neural community education. To further improve the model’s overall performance, a dual-augmented attention mechanism was incorporated. This method contains spatial and temporal attention layers, permitting the version to dynamically focus on the maximum huge factors even as nonetheless shooting complex relationships in the EEG information. The model can effectively categorize EEG signals by combining ResNet’s study characteristic extraction with better interest mechanisms, taking use of each residual connection and refined attention to maximize overall performance and reliability. The ResNet model is enhanced with spatial and temporal attention mechanisms. The incorporation of spatial and temporal attention layers into ResNet may be elucidated by specifying their architectural positioning and operational roles. Spatial attention layers prioritize essential EEG channels by creating attention maps from intermediate convolutional feature outputs, which are then multiplied element-wise with feature maps to enhance significant areas. Temporal attention layers, conversely, allocate weights to various time periods in EEG data, emphasizing crucial parts that represent brain activity. Attention techniques may be included at several junctures within the ResNet architecture, including between residual blocks or after feature extraction, thereby enhancing concentration on relevant characteristics. The Reptile Search Algorithm (RSA) is used for feature selection because of its strong capacity to balance exploration and exploitation, efficiently traversing high-dimensional EEG feature spaces. RSA’s hunting-inspired technique facilitates dynamic optimization, surpassing standard methods such as Particle Swarm Optimization (PSO) and Genetic Algorithms (GA) in evading local optima. By choosing the most relevant features from the autoencoder output, RSA enhances the input to ResNet, hence augmenting classification accuracy. Direct comparisons with other optimization methods would further validate the choice of RSA and its incorporation into the feature selection process, then improves ADHD classification by dynamically focusing on the most relevant features of EEG data. Spatial attention prioritizes key EEG channels that capture critical patterns indicative of ADHD, allowing the model to amplify important regions while suppressing irrelevant ones. Temporal attention, on the other hand, highlights significant time intervals where variations in EEG signals reflect crucial neural activity related to ADHD. Together, these mechanisms enable the model to efficiently extract both spatial and temporal features, ensuring a comprehensive analysis of the EEG signals. The integration of these attention mechanisms with ResNet’s residual learning capabilities helps the model overcome challenges like vanishing gradients, leading to more accurate and robust ADHD classification.

The classification model utilized a modified ResNet featuring a dual-augmented attention mechanism.

Architecture: The ResNet model was tailored for EEG signal classification with modifications including the following:

Layer Configuration: Eighteen residual layers featuring skip connections.

Kernel Sizes: One-dimensional convolutional layers utilizing kernel sizes of 3, 5, and 7 to capture both short- and long-term signal relationships.

Stride and Padding: A stride of 2 facilitated downsampling, whereas same-padding maintained feature dimensions.

Double-Augmented Attention Mechanism integrates two complementary attention components:

Spatial Attention: Concentrated on essential EEG channels.

Temporal Attention: Emphasized significant time periods.

### 3.9. Model Evaluation

The method of “version evaluation” examines whether a produced model may be implemented to fresh information to ascertain the model’s generalizability. Several key metrics are employed to evaluate categorization performance, including accuracy, precision, recall, F1 score, specificity, and sensitivity. Iterative validation methods, such as cross-validation, rigorously refine these estimates, ensuring their robustness and reliability. This approach enriches evaluative tools, such as confusion matrices, with extensive statistical insights. Comprehensive assessment plays a crucial role in refining models for both development and practical deployment.

#### ResNet

**Figure 3:**
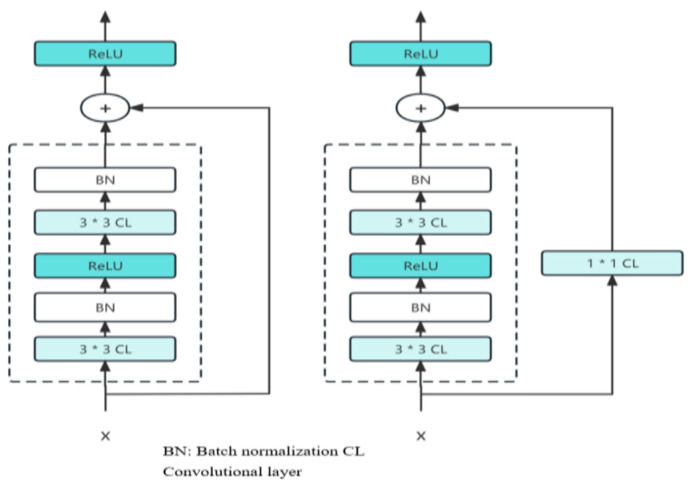
Key structure of ResNet.

Recurrent Neural Network (ResNet) is a network architecture that builds upon the VGG19 network by incorporating residual units via a short link mechanism. The challenge of performance attenuation, wherein accuracy diminishes with an increase in network depth, is alleviated through the incorporation of identity mapping, represented by curved lines and residual mapping, characterized by the model’s residual components. These methodologies safeguard the preservation of informational fidelity as it traverses through successive layers of greater depth. ResNet implements the full 3 × 3 convolutional layer architecture of VGG. It incorporates a Batch Normalization layer and a ReLU activation function. Additionally, it introduces an additional 1x1 convolutional layer to convert the input into the exact shape required. This transformation is then directly combined with the residual function output. Reducing the feature map size by half doubles the size of the map, as shown in the model, which is a basic design element of ResNet. The complexity of network levels may be preserved with the help of this notion. To include residual learning, the ResNet design introduces a short connection mechanism across every two layers. Their main differences are that ResNet uses stride = 2 convolution for down sampling and that a global average pool layer replaces the fully connected one. The key structure of ResNet is shown in Figure 3.

Through its chosen approach, the implementation of zero-padding results in an increase in dimensions and eliminates the occurrence of arguments within the network. An alternative approach would be to implement a novel mapping technique called the projection shortcut, which typically involves a 1 × 1 convolution. This method results in an increase in parameters and hence raises the computational workload.

### 3.10. Performance Metrics

Accuracy: The simplest way to measure how often the classifier makes correct predictions is by using accuracy. Another way of looking at it is that this is the percentage of correct forecasts relative to all guesses.

(7) $$Accuracy=\frac{TP+TN}{S}.$$

Precision: In contrast to this ratio, in addition subtracting it from one minus, i.e., (1 − precision), which represents the percentage of false negatives. Additionally, recall can be derived as the inverse of precision, offering a distinct perspective on classification performance.

(8) $$Precision=\frac{TP}{TP+FP}.$$

Recall: On the other hand, there are so-called false negatives in relation with True Negatives.

(9) $$Recall=\frac{TP}{TP+FN}.$$

F1-Score: It is calculated by taking the accuracy and recall scores and squaring them.

(10) $$F1=\frac{2*Precision*Recall}{Precision+Recall}.$$

AUC Calculation: The AUC is computed as a graph of the ROC curve, describing the relationship between TPR and FPR for different thresholds. The value of the AUC corresponds to the area below the curve. Values close to 1.0 indicate high performance, and a higher AUC generally implies that the model can distinguish between classes more accurately. The AUC can be calculated using the formula given below.

(11) $$AUC=0.5*\left(TPR+TNR\right)*100.$$

Specificity: The ability of the model to correctly identify negative cases. Specificity measures the ratio of actual negative cases that are correctly identified. It is calculated as follows:

(12) $$Specificity=\frac{TN}{TN+FP}$$

Sensitivity: The ability of the model to correctly identify positive cases. Sensitivity measures the ratio of actual positive cases that are correctly identified. Sensitivity is also called Recall. It can be calculated as follows:

(13) $$Sensitivity=\frac{TP}{TP+FN}$$

Table 2 shows the specificity and selectivity of all the models. A high sensitivity proposed model indicates that the model can detect most of the relevant cases, making it crucial in applications compared to Adaboost and Random Forest model. On the other hand, high selectivity for the proposed ResNet model ensures that the model minimizes false cases by accurately excluding irrelevant or negative cases compared to Random Forest and Adaboost.

The EEG data used in this study underwent a variety of preprocessing steps. After the dataset was analyzed, class labels were saved independently to confirm the distribution of classes between the “normal” and “ADHD” categories. Following the 10–20 system montage, the MNE library was used to prepare the EEG data. The channel names were acquired, and an info object with the sample frequency and EEG channel data was produced. The normal and ADHD groups’ data were then extracted from the DataFrame and converted to NumPy arrays. After that, these arrays were reversed so that channels appeared as rows and samples as columns. These arrays were converted into MNE RawArray objects for further processing. For outlier treatment, artifacts in EEG facts were diagnosed and removed the use of ICA. The EEG facts were cleaned by identifying and getting rid of ICA additives connected to the artifacts. Plotting the cleaned records allowed us to affirm that the artifacts had been successfully removed. Reptile Search Algorithm (RSA) is an optimization algorithm for the selection of most relevant features from the EEG dataset for feeding into a machine learning model. Specifically, it is used to solve the feature selection problem. This is common when dealing with high dimensional datasets, such as EEG data, which often has a large number of features (channels in this case). Not all the features contributing to the task are meaningful (i.e., separating ADHD from normal subjects). Too many features overfitting and a quick increase in computational complexity may even decrease performance. Feature selection methods are typically developed to select the most relevant subset of features with the best predictive performance.

## 4. Results and Discussion

This study included 121 participants (61 with ADHD and 60 without), providing a dataset of 50,000 samples. The focus of this study was to identify a dataset with greater variability and a balanced representation of ADHD and healthy subjects. The process included building a model using a ResNet architecture that was tweaked with a double-augmented attention mechanism, feature selection employing the Reptile Search Algorithm, and feature extraction with an autoencoder. Various evaluation metrics have been employed for this study, including F1 score, accuracy, precision, and recall, to assess the efficacy of the classification model. This method has the ability to improve ADHD diagnosis by using EEG data processing.

**Figure 4:**
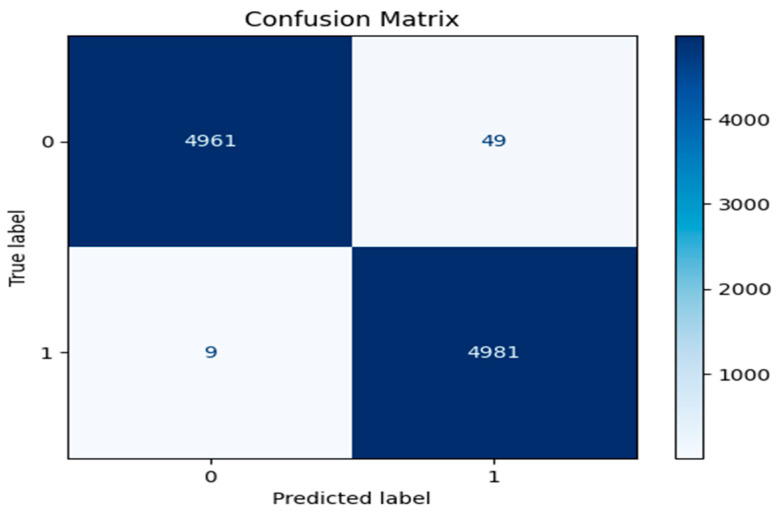
Confusion matrix for ResNet with Double Augmented Attention Mechanism.

Figure 4 shows ResNet with double Augmented attention module confusion matrix with exceptional model performance. Out of 10,000 samples, the model demonstrates exceptional performance with 4961 records correctly identified as class 0 (True Negatives) and 4981 records accurately classified as class 1 (True Positives). Misclassifications are minimal, with only 49 False Positives (class 0 misclassified as class 1) and 9 False Negatives (class 1 misclassified as class 0), signifying a highly precise model.

**Figure 5:**
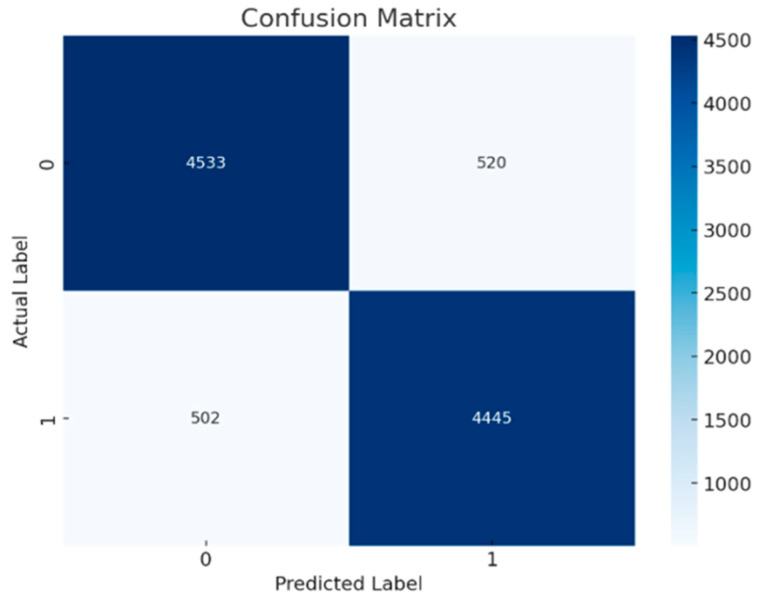
Confusion matrix for Random Forest classifier.

**Figure 6:**
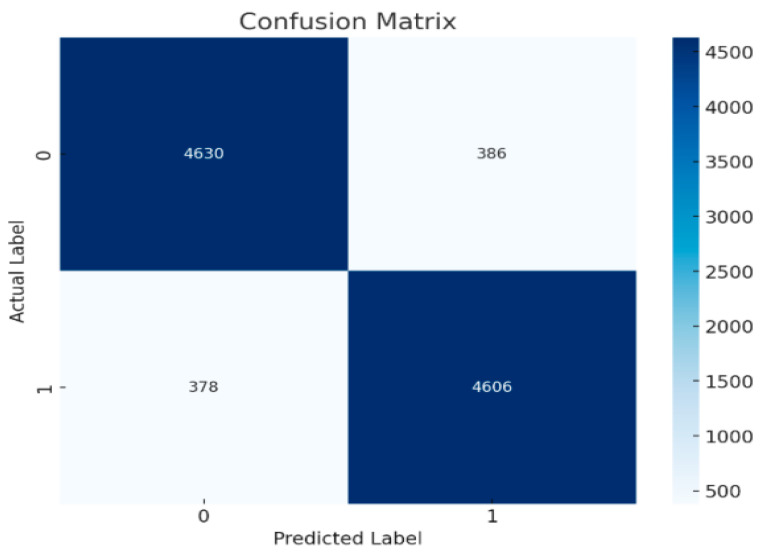
Confusion matrix for Adaboost classifier.

Figure 5 shows the Random Forest confusion matrix with a slight decline in accuracy compared to the ResNet with double Augmented. It records 4630 True Negatives and 4606 True Positives, with misclassifications increasing to 386 False Positives and 378 False Negatives, indicating a moderate decrease in performance. Similarly, Figure 6 shows the confusion matrix of Adaboost classifier, further highlighting an escalation in misclassifications. It records 4533 True Negatives and 4445 True Positives, with 520 False Positives and 502 False Negatives, reflecting a more noticeable decline in accuracy compared to the AdaBoost results.

According to the obtained results, ResNet has the highest accuracy with minimum misclassifications. AdaBoost and RandomForest, however, have some considerable increases in the rate of misclassifications that could, in fact, represent some true degradation in the performance of the model. The three matrices illustrate differing levels of model performance, with the ResNet with double Augmented attention model exhibiting the highest efficacy, whereas the others demonstrate elevated misclassification rates, underscoring the necessity of error reduction to enhance prediction accuracy. It would be possible to make a judgment on whether these differences in performance are statistically significant if the misclassification rates and the overall accuracy are examined. Since there were variations in True Positives, True Negatives, False Positives, and False Negatives between the three models, the differences in performance are likely to be statistically significant. The misclassification rate for all the models is shown in Table 3.

Misclassification rate = FP + FN/(TP + TN + FP + FN).

**Figure 7:**
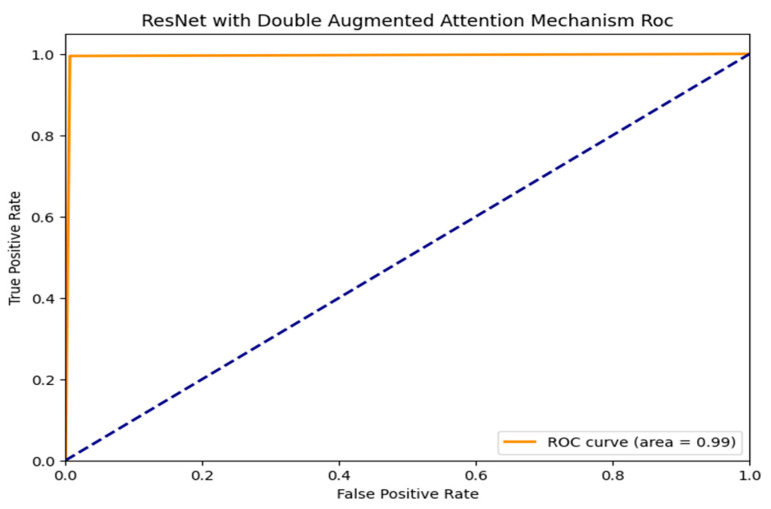
ROC_AUC curve for ResNet with Double Augmented Attention Mechanism.

**Figure 8:**
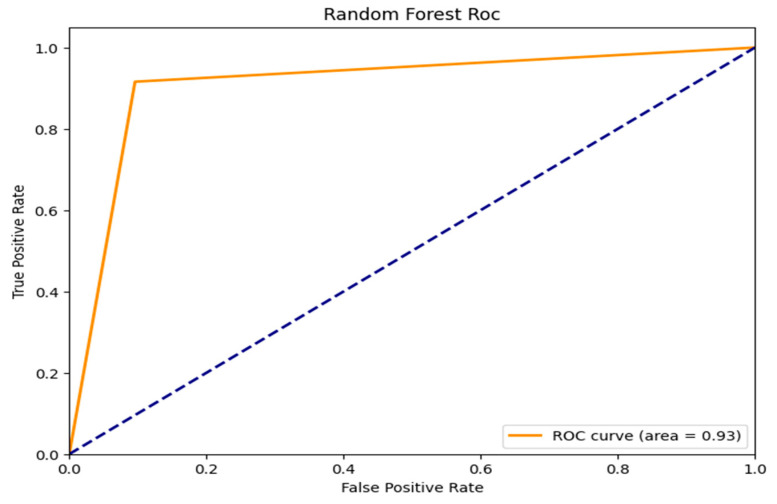
ROC_AUC curve for Random Forest Classifier.

**Figure 9:**
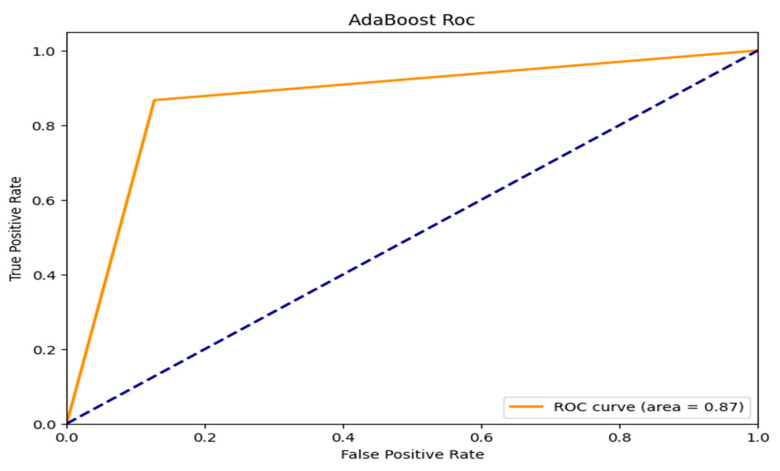
ROC_AUC curve for Adaboost Classifier.

The ROC curves for three machine learning models, which are evaluated based on their ability to distinguish between classes, Random Forest, AdaBoost, and ResNet with Double Augmented Attention Mechanism, are shown in Figure 7, Figure 8 and Figure 9. As can be seen in Figure 7, ResNet with Double Augmented Attention Mechanism performs very well, whose ROC curve nearly hugs the upper left corner. With an AUC of 0.99, the classification skill is almost flawless. The model detects genuine positives with almost no misclassification, given the steep climb of the curve and the low False Positive Rate. With an AUC of 0.93, as shown in Figure 8, Random Forest also performs strongly and shows good prediction accuracy, as indicated in Figure 8. However, the ROC curve is not as steep as that of the ResNet model’s, and it shows that Random Forest is slightly less accurate than ResNet, although still good at class distinction. The TPR and FPR are well-balanced in the model. AdaBoost performs excellently, as shown in Figure 9, with an AUC of 0.87. However, it still shows the ability to distinguish between the classes, although its curve is not as prominent as the first two models. Although the model does well, it does not compare to Random Forest and ResNet. All the models perform better than random guessing, which is shown by the dashed diagonal line. However, ResNet has the best classification.

**Figure 10:**
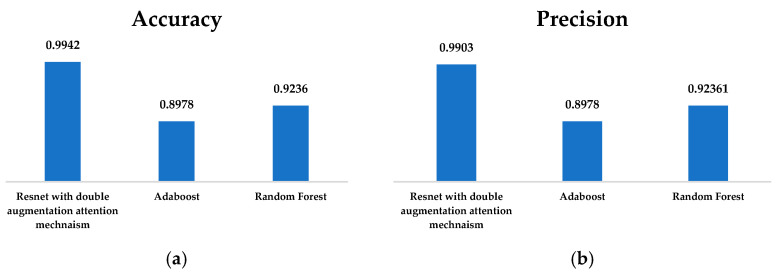
(**a**) Accuracy and (**b**) precision of ResNet with Double Augmentation Mechanism, AdaBoost, and Random Forest Classifier.

**Figure 11:**
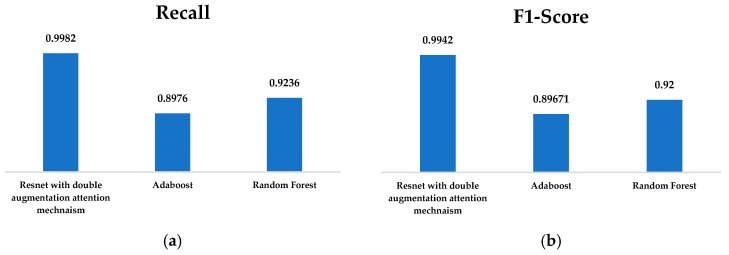
(**a**) Recall; (**b**) F1-Score of ResNet with Double Augmented Attention Mechanism, AdaBoost, and Random Forest Classifier.

Some of the three models—RandomForest, AdaBoost, and ResNet with double augmented attention module— had a higher level of predictiveness in their respective performance metrics. The highest F1-score, that is, 0.9942 accuracy value of 0.9942, precision value of 0.9903, and recall value of 0.9982, shown Figure 10 and Figure 11, was obtained by the model with the double-augmented attention module, ResNet. This indicates that the model is very good at producing high-quality predictions and has an impressive capacity to identify real positives, as indicated by its almost flawless recall. The presence of the double augmented attention module probably improves the ability of the model to concentrate on relevant features, which boosts classification accuracy. AdaBoost produced some average results; F1-score was 0.8967, recall score was 0.8976, and accuracy and precision were 0.8978, as shown in Figure 10 and Figure 11. These scores, in comparison to the ResNet-based model, result in the observation that AdaBoost is not very resistant but still fairly successful. Although it combines several weak classifiers into building a strong one, the low recall rate shows that it may miss some of the actual positives, leading to its total performance. RandomForest surpassed AdaBoost with an F1-score of 0.92, accuracy of 0.9236, precision of 0.92361, and recall of 0.9236, as shown in Figure 10 and Figure 11. But the results were short of those of ResNet. It is the ensemble method, and it uses many decision trees to build a model. It has good generalization. Thus, it reduces overfitting. Its much lower F1-score depicts little disparity in the recall and precision. Because the ResNet model with two-fold enhanced attention module has its deep learning structure and sophisticated attention mechanism, it is perfect for challenging jobs. On the other hand, RandomForest and AdaBoost have respectable but somewhat limited predictive capabilities, making them appropriate for simpler datasets or situations where computational speed is an issue.

Table 4 shows the comparisons of the model’s performance on important metrics and *p*-value results. The proposed model, ResNet with double Augmented Attention, outperforms the other models—AdaBoost and Random Forest—in all four important metrics: Accuracy, Precision, Recall, and F1-Score. It is clear that there are considerable disparities in performance, with ResNet clearly demonstrating much better results across the board. The results of the statistical analysis indicate that the ResNet (*p* < 0.001) is statistically significant. This further substantiates the superiority of the proposed model. The comparison of AdaBoost and Random Forest, on the other hand, reveals that there is no significant difference (*p* > 0.05), indicating that their individual performances are comparable. As a result, ResNet with double Augmented Attention is unquestionably the best model, since it has a large advantage over the other two models in all of the criteria that were analyzed.

According to the basic data, ResNet with double Augmented Attention performs better than the other models when it comes to accuracy, precision, and memory. All models were analyzed on 95% confidence interval. Table 5 shows the comparisons of model’s performance respect to mean, standard deviation, lower bund, and upper bound. ResNet achieves an average accuracy of 0.9946 (SD = 0.00597), a mean precision of 0.9895 (SD = 0.00444), and a mean recall of 0.9977 (SD = 0.00643). The confidence ranges for all of these measurements are very small. Random Forest and AdaBoost, on the other hand, do not do as well, with mean accuracies of 0.9230 and 0.8972, respectively, and similar trends in precision and recall. Overall accuracy, precision, and recall scores for all models are all around 0.938, further supporting the conclusion that ResNet is the best model for this task.

The effects of these studies suggest that the proposed ResNet model with a double-augmented attention mechanism is a success at detecting ADHD using EEG statistics. The model’s exceptional ability to differentiate between children with and without ADHD is demonstrated through its outstanding precision, recall, and F1-score metrics. The robustness of the ResNet model is further validated by its comparative performance against alternative models, such as AdaBoost and Random Forest. While AdaBoost and Random Forest fared properly, their lower ratings throughout numerous classes suggest that algorithms are less powerful at detecting subtle patterns in EEG statistics.

The confusion matrix shows that the ResNet version not only reduces misclassifications but also excels at efficaciously identifying authentic positives and actual negatives. The misclassification rates of 0.58% for the ResNet, 10.22% for AdaBoost, and 7.64% for Random Forest highlight significant clinical relevance, particularly in diagnostic scenarios where accuracy directly impacts patient outcomes. A misclassification rate as low as 0.58% suggests the proposed model’s potential for high reliability, reducing false diagnoses that could lead to unnecessary or incorrect treatments. This level of precision is especially critical for conditions like ADHD, where accurate early detection can significantly influence therapeutic decisions and long-term patient management. The ResNet model’s capacity to retain high overall performance while producing few false positives and negatives suggests that it might be an effective diagnostic device for ADHD.

Furthermore, the ROC AUC value of 0.99 demonstrates that the ResNet version exhibits exceptional discriminating abilities, confirming its superiority over other models. The ResNet model’s close to-perfect ROC AUC price demonstrates its high-quality sensitivity and specificity, organizing it as a reliable model for ADHD classification.

To conclude, the ResNet model with a double-augmented attention mechanism outperforms present ADHD type methods together with AdaBoost and Random Forest. These findings imply that incorporating State-of-the-Art attention processes into deep learning models could substantially enhance their potential to acquire and interpret complex biological statistics, potentially leading to more accurate and reliable diagnostic tools in medical practice.

## 5. Conclusions and Future Scope

Using electroencephalogram (EEG) statistics and advanced machine learning algorithms, this research concludes with a completed technique for ADHD category. The proposed ResNet model achieves better outcomes than top-tier models like Random Forest and AdaBoost across numerous metrics, including F1 rating, recall, accuracy, and precision, due to its double-augmented attention mechanism. Based on its near-optimal ROC AUC rating, the version has a low misclassification charge and excellent sensitivity and specificity, which would make it a powerful tool for ADHD diagnosis.

Given that the ResNet model was able to discover intricate patterns in EEG facts, it is clear that attention processes must be coupled with deep learning for biological applications. In medical contexts, the ability of faulty diagnoses is a major concern; this strategy complements the device’s classification accuracy while addressing this issue. The findings of this study imply that the proposed model has the capability to be used in practical conditions, supplying medical professionals with a tool for diagnosing ADD/ADHD appropriately and at an early level.

With an emphasis on the ability of deep learning models with attention mechanisms to enhance diagnostic procedures, this study adds to the expanding body of information about the use of machine learning in healthcare. Additional research might look at using this method for other types of neurological illnesses, which would prove its usefulness and increase its impact on medical diagnosis.

This study also has its limitations. A visual attention task, which focuses on a single component of attention, served as the basis for the EEG recording procedure. Working memory, executive function, auditory attention, and other cognitive processes are all impacted by ADHD, which is a complex condition. The limited emphasis on visual activities may not offer a thorough comprehension of the brain activity associated with ADHD. Ritalin had been used by some children diagnosed with ADHD for as long as six months. The medication may introduce confounding variables by affecting EEG readings and brain activity. It may be difficult to extrapolate the results to children with ADHD who are not taking Ritalin due to its effects on EEG patterns.

Given these constraints, it would be advantageous to develop EEG recording protocols that incorporate a greater variety of cognitive activities, including working memory, inhibitory tasks, auditory and executive attention, and others, in order to fully capture the breadth of cognitive functioning and impairments associated with ADHD. In order to properly separate the brain markers of ADHD without the influence of pharmacological therapies, future research should consider children with ADHD who have not taken any medication; this would make it easier to distinguish between changes caused by medication and brain activity related to ADHD. A more thorough knowledge of the neurological correlates of ADHD might also be possible by extending EEG recording to additional brain networks and regions implicated in the disorder, such as the frontal-lobe circuits linked to executive functions.

The future aim is to augment the capabilities of advanced deep learning model to develop an application-based system that will aid psychiatrists by integrating ML/DL-powered tools for the automation and optimization of ADHD symptom assessment.

## Figures and Tables

**Table 1:** Models related to ADHD classification using Deep learning for various datasets.

Author	Year	Dataset	Model, Validation Method	Accuracy	Recall	Precision	F1 Score	ROC_AUC
Chen M et al. [17]	2019	977 participants	mcDN Hold-out	-	-	-	-	0.82
Dubreuil-Vall L. et al. [18]	2020	40 participants, comprising 20 healthy adults (10 males and 10 females) and 20 adults with ADHD (10 males and 10 females)	CNN, LPOCV	88%	-	-	-	-
Tosun et al. [19]	2021	EEG Data	LSTM, No	92.15%	-	-	-	-
Zhou et al. [20]	2022	Children of 6–16 years	CNN, No	97.7%	-	-	-	-
Donglin Wang et al. [21]	2022	-	CNN No	69%	-	-	-	-
Niang Qiang [22]	2022	-	Spatiotemperal atttention autoencoder No	72.5%	-	-	-	-
Jungpil [23]	2023	156 samples from ADHD children with coexisting ADD and 180 samples of TD children	CNN LOOCV	94%	89.7%	97.8%	91.3%	0.938
Mustafa Yasin Esas et al. [24]	2023	EEG data obtained from children aged 7 to 12 years, comprising 61 individuals diagnosed with ADHD and 60 age-matched controls without ADHD	CNN CV	87%	-	-	-	-
Miguel Garcia Argibay et al. [25]	2023	238,696 individuals residing in Sweden during the period from 1995 to 1999	DNN No	69%	71.6%	65.0%	-	0.75
Wonjun Lee et al. [26]	2023	Screen video game	LSTM GRU RNN No	97.82% 96.81% 96.81%	-	-	-	-

**Table 2:** Model Evaluation using specificity and selectivity.

Model	Specificity	Sensitivity
ResNet with Double Augmented Attention Mechanism	0.99	0.99
Adaboost	0.89	0.89
Random forest	0.92	0.92

**Table 3:** Misclassification Rate Calculation.

Model	False Positive	False Negative	Misclassification
ResNet with Double Augmented Attention Mechanism	49	9	0.58%
Adaboost	520	502	10.22%
Random forest	386	378	7.64%

**Table 4:** Comparing Models’ Performance on Important Metrics with *p*-Value.

Model	Accuracy	Precision	Recall	F1-Score	*p*-Value
ResNet with double Augmented Attention	0.9942	0.9903	0.9982	0.9942	0.0005
AdaBoost	0.8978	0.8978	0.8976	0.8967	0.08
Random Forest	0.9236	0.9236	0.9236	0.92	0.06

**Table 5:** Comparing Models’ Performance on Important Metrics with Statistical Significance.

		N	Mean	Std. Deviation	95% Confidence Interval for Mean	
					Lower Bound	Upper Bound
Accuracy	ResNet with double Augmented Attention	20	0.9946	0.00597	0.9918	0.9974
	AdaBoost	20	0.8972	0.00473	0.8950	0.8995
	Random Forest	20	0.9230	0.00485	0.9207	0.9253
	Total	60	0.9383	0.04186	0.9275	0.9491
Precision	ResNet with double Augmented Attention	20	0.9895	0.00444	0.9874	0.9916
	AdaBoost	20	0.8968	0.00593	0.8940	0.8996
	Random Forest	20	0.9238	0.00459	0.9216	0.9259
	Total	60	0.9367	0.03958	0.9265	0.9469
Recall	ResNet with double Augmented Attention	20	0.9977	0.00643	0.9947	1.0007
	AdaBoost	20	0.8959	0.00431	0.8939	0.8979
	Random Forest	20	0.9211	0.00449	0.9190	0.9232
	Total	60	0.9382	0.04396	0.9269	0.9496

## Data Availability

The raw data supporting the conclusions of this article will be made available by the authors upon request to the corresponding author due to ethical reasons.
